# 

**Published:** 1879-02

**Authors:** 


### MISSISSIPPI VALLEY DENTAL ASSOCIATION.

The thirty-fifth annual meeting of the Mississippi Valley Dental Asssociation will take place in Dental College Hall, Cincinnati, O., on the first Tuesday in March, 1879, at 2 o'clock p. m.

OFFICERS.

President, Dr. Frank A. Hunter.

First Vice President, Dr. J. S. Cassidy.

Second Vice President, Dr. J. W. Jay.

Recording Secretary, Dr. E. G. Betty.

Corresponding Secretary, Dr. C. I. Keely.

Treasurer, J. G. Cameron.

The following are the

SUBJECTS FOR DISCUSSION.

1. Chemical Processes that Occur in the Mouth.
2. Effects of Systemic Diseases upon the Dental Organism.
3. The Influences upon Second Dentition of the Derangement of the First.
4. The New Dental Departure.
5. Dental Education and Professional Culture.
6. Novel Dental Operations.
7. Modus Operandi of Topical Remedies in Inflammatory Conditions of the Dental Organism.
8. Morbid Conditions of the Pulp, and how Diagnosed.

A full attendance at the opening of the meeting is earnestly requested.

All members of the profession, and others interested in the advancement of dental science, are cordially invited to attend.

### CALIFORNIA STATE DENTAL ASSOCIATION.

The tenth annual session of the California State Dental Association will be held in San Francisco, commencing Tuesday, May 13th, 1879, at 10 o'clock a. m., and continue four days.

SUBJECTS OF PAPERS.

Reflex Influence of Dental Associations and Dental Colleges on the Dental Profession. W. H. Robinson.

Legislation Pertaining to Dentistry. H. J. Plomteaux.

Conservative Dentistry. R. Cutlar.

Periostitis. Cyrus Hamilton.

Pulpitis and its Treatment. J. A. W. Lundborg.

Dental Legislation. C. E. Davis.

Professional Self-reliance. Jno. Rabe.

Professional Ethics. J. J. Birge.

The Wages of Professional Intercourse. Wm. B. Kingsbury.

Mechanical Dentistry Defined. W. C. Harding.

Oral Electricity. J. L. Cogswell.

Professonal Culture. G. L. Jenkins.

Ulitis. C. S. Lane.

### MEETING.

The Ohio Dental College Association will hold the annual meeting on Tuesday, March 4th, at ten o'clock a. m.

It is very desirable that the members be present so far as possible, and take part in the deliberations, and do whatever may be required for the interest and prosperity of the college.

Let all be promptly in attendance, for the succeeding days will be very fully occupied with other things.

### COMMENCEMENT.

The annual commencement exercises of the Ohio Dental College will be held at half past seven o'clock p. m., Wednesday, March 5th, to which all the friends and those interested in the college are most cordially invited.

### *THE DENTAL REGISTER,*

A MONTHLY MAGAZINE DEVOTED TO THE INTERESTS OF

THE DENTAL PROFESSION.

*Terms Reduced to $2 50 per Year. Single Copies 25c.*

*EDITED BY PROF. J. TAFT, D. D. S.,*

AND PUBLISHED BY

SPENCER & CROCKER, Ohio Dental Depot.

The volume commences in January and closes in December.

The Register being issued monthly is always fresh, therefore Indispensable to the practitioner who desires to keep posted up with the new inventions and improvements which are daily developed. Neither expense nor effort will be spared to give value and variety to its contents. Articles will be illustrated with well executed engravings whenever they will add to the interest or assist to explanation.

Its extensive circulation and frequency of issue make it one of the BEST DENTAL ADVERTISING MEDIUMS in the United States.

All subscrptions must begin with January or July.

Local or special notices, five lines of less, will be inserted for $3.00 each insertion; over five lines, 50 cts. per line.

All advertising bills payable in advance, or at furthest, after the issue of the first number containing the advertisement. All transient advertisements to be paid for in advance.

☞CONTRIBUTIONS SOLICITED.

Exclusively Editorial Communications should be addressed to Editor.

The latest number of the Register may be ordered with or without other goods at any time, and all letters should be addressed to

**SPENCER & CROCKER, Ohio Dental Depot, Cincinnati, O.**

